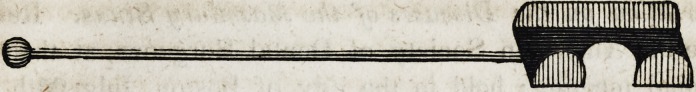# A Treatise on Mechanical Dentistry

**Published:** 1842-12

**Authors:** Solyman Brown


THE AMERICAN

### JOURNAL OF DENTAL SCIENCE.

Vol. III.] DECEMBER, 1842. [No. 2.

### ARTICLE I.

*A Treatise on Mechanical Dentistry.* By Solyman Brown, M. D., D. D. S.

(Continued from page 279, v. ii.)

### CHAPTER IV.

*Of Double Sets with Springs.*

84. The writer has the satisfaction to learn, that, in the three preceding chapters he has had the singularly good fortune to be so unreserved, minute, and particular in his developments of what some pretend to consider *the secrets of the art,* as to incur the displeasure of a certain class of his professional brethren who seem to be afraid of making knowledge so common as to give it the character of an *epidemic.* It will be the unremitted endeavour of the author of this treatise, to deserve hereafter the reiterated rebuke of this portion of the profession, by entering if possible, still more deeply into the mysteries which they would selfishly conceal. He is resolved never to become the abettor of that species of philosophy which pretends to consult the welfare of mankind by concealing useful knowledge; and which fain would humiliate a noble profession by allying it to the mysteries of quackery. The day has gone by, when a voluntary contributor to a scientific periodical shall be brow-beaten into either silence or concealment; and the subscribers to this journal may rest assured that while the author of this treatise is a contributor to its pages, "*the truth, the whole truth,*" and (if possible) "*nothing but the truth,*" shall be regularly communicated to the profession, in regard to all improvements in our art, as fast and as far as they come to the knowledge of the writer. His constant intercourse with the most distinguished members of the profession, as well verbally as by letter, in all parts of the United States, enables him to compare their different modes of practice, and to embody in these chapters those methods which he esteems the best; and this he shall continue to do "*without the fear or the favour*" of any living man. He may peradventure be removed from the editorial chair of "the American Journal and Library of Dental Science," for the independence of his course in this behalf—which indeed has been already attempted without success; but this will neither intimidate him in his duty, nor divert him from his course. He seeks neither office nor honour from any man, or from any set of men, who would subject him to the humiliating condition of retaining his place by suppressing information which is useful to mankind.

85. In the three former chapters of this treatise, the student has been conducted by a single path selected from among many almost equally frequented, to that part of our subject which treats of what are appropriately denominated in the vocabulary of our art, "*double sets of teeth with springs.*"

This apparatus so necessary for many important purposes to those individuals who have lost all or nearly all their natural teeth, is capable of being constructed with so much neatness and durability as to render them almost an adequate substitute for the natural organs of mastication. Genius of a high order, and industry untiring have been long devoted to the improvement of these double sets, insomuch that it is now difficult to imagine how the ingenuity of posterity can superadd many improvements to the present effectual methods of construction.

86. The difficulties to be overcome in solving the practical problem of constructing a permanent, beautiful and efficient apparatus to be used in the place of natural human teeth, have been neither few nor small; and the consequence has been that hundreds, nay, thousands of years have been employed in its satisfactory solution. We have no evidence that the ancients ever carried the art of dentistry so far as to furnish to any individual, an entire double set of artificial teeth; and the efforts even of the moderns, have been ineffectual in fully supplying the demand in all its reasonable requirements, until the present century. The conditions of the problem are as follows:—

*First*—That the materials employed in all parts of the fabric, shall be, to a reasonable extent, indestructible either by the mechanical action of the parts upon each other, or by the fluids of the mouth.

*Secondly*—That the entire apparatus shall have the appearance of the natural teeth, so that the substitution shall not be evident to the beholder.

*Thirdly*—That the artificial teeth shall restore the articulation of language to its original condition.

*Fourthly*—That they perform the function of mastication so as to secure healthy alimentation to the physical system.

*Fifthly*—That the whole of these objects be attained with the smallest possible amount of pain and inconvenience to the patient.

*Sixthly*—That the expense of the apparatus be limited to the ability of the industrious as well as the wealthy classes of community.

To accomplish all these objects, has required many years, many experiments, and much study; and until the modern invention of incorruptible, mineral teeth, the solution of the problem seemed to elude the sagacity of man. But at length Art has triumphed, for Genius has come to her aid; and the generation which travels by steam on the land, the ocean and the air; which extinguishes the chivalry of warfare by the use of steam-ships, chambered rifles, and Paixhan shot; and which augments the pleasures of life by an interchange of commodities with every continent, island, and climate of the earth, has reserved to itself the privilege and the pleasure of restoring to man his lost mandibles even after their utter destruction.

87. I have already intimated that there are many distinct modes of arriving at the same object in mechanical dentistry; and it shall be my endeavor first to describe what I esteem, on the whole, the most convenient method of constructing and adapting double sets, and afterwards to lay before the student other methods by which the same object may be attained with equal success in the result, if not with equal facility in the execution.

88. When a case presents itself where a double set is evidently required, the patient, if a novice in matters relating to this species of manufacture, should be fully and distinctly apprised of all the difficulties and inconveniences to be encountered in bringing such an apparatus into successful operation. The fact that so great a change as that from bare gums to a full set of teeth, must at first embarrass the articulation, should be distinctly impressed on the memory of the patient; for he will generally forget at least four-fifths of all you say respecting the difficulties to be overcome and the inconveniences to be endured in becoming accustomed to whole sets of artificial teeth. You should state emphatically that, in all cases, more or less irritation and consequent inflammation will attend the introduction of such pieces into the mouth, just as the friction of new shoes upon the feet, or of the strings of a harp upon the fingers, will of necessity result first in irritation and afterwards in induration of the parts. The gums must become in some degree callous before they cease to be sensitive under the pressure attendant upon the operation of masticating food with artificial teeth. Let it be stated moreover that the adhesion of plates to the subjacent parts, is not at first so perfect as it will be after a few weeks' use. When all these facts have been properly explained and inculcated, and when the patient in full view of them, is willing to encounter the necessary temporary inconvenience for the sake of future and ultimate advantage, the way is prepared for a preparation of the mouth for a double set.

89. If some of the front, lower teeth remain in a healthy state, which is often the case when double sets with springs are required, let them be thoroughly cleaned, so that the match may be correct when the artificial teeth are selected, and in all cases let every remaining root of the natural teeth be extirpated, leaving a clean and healthy gum. Too much time is rarely allowed for the healing of the gums and simultaneous absorption of the alveoli, after removing the old teeth and roots preparatory to the introduction of a double set. The period required varies so exceedingly in different cases, that no definite rule can well be established; a little experience and observation will be the best instructors.

90. When the mouth is found to be well prepared, wax impressions may be taken of both jaws, as has been already described when treating of upper and lower sets. The wax should extend well back into the mouth and embrace the alveolar ridge completely, spreading over most of the palatal arch of the superior maxillary, extending down to the sublingual muscles of the lower jaw, and in all cases covering as much of the outer surfaces of both the alveolar ridges as the muscles and integuments of the cheeks will permit. At least two wax impressions of each jaw must be thus procured, with the utmost accuracy and care, because the excellency of the fit constitutes a great charm in a double set of artificial teeth. When these wax impressions have been slightly oiled let one of each jaw be filled with pure plaster and the other with plaster mixed with sand and powdered charcoal, in the proportion of one-half plaster, one-fourth sand and one-fourth charcoal; let all these casts be well dried and they are ready for use, those of pure plaster for obtaining metallic castings, and those of mixed ingredients for sustaining the work when subjected to heat in the act of soldering. This mixture of plaster, sand, and charcoal, constitutes, I believe, the best known composition for this purpose, not only because of its little liability to crack under the blowpipe, but because the conducting power of the charcoal being small, the heat approaches the enamel of the teeth in a gradual manner which secures them from the danger of being cracked, and moreover the partial combustion of the charcoal augments the heat and hastens the operation.

91. After the metallic castings have been obtained from those in plaster, various methods are pursued by different individuals. As I have already described the method of taking metallic casts by many distinguished operators, I shall now introduce that long since adopted by Dr. Harris, of Baltimore, and perhaps by others of the profession, and indeed which I myself now prefer, after several experiments, to any other with which I am acquainted. Take a cast iron ring, the hub-box of a cart or waggon wheel, for example, or a sheet iron rim of the following shape, and a little larger at one end than at the other. Fit into this ring or rim a sheet iron, tin, copper or zinc shelf that just fills it at about the centre of its altitude, as at B. Cut the plaster cast as thin as convenient, taking care to leave all that portion on which you desire the gold plate ultimately to rest. Set the flattened side of the plaster cast upon the shelf B, while the opposite end of the ring is immersed in a cold sand bath, so as to keep the shelf in its proper position, and also to prevent any of the melted metal from escaping between the shelf and the ring. The surface of the plaster cast should be fitted very nicely to the shelf in order to prevent the metal from running between them, for which purpose I use a grater of the following construction. Into a wooden box fourteen inches in length, four inches wide and four inches deep insert a tin plate grater about an inch from the top, resting upon cleets to preserve it in its place and keep it level. Upon this simple machine the plaster cast may be made quite smooth in a few seconds. The cast thus prepared must be held down firmly upon the shelf by the aid of some iron instrument of small size, until the melted metal which is poured upon it shall begin to consolidate about the sides, whereupon the iron should be withdrawn. In a former part of this treatise I have recommended lead and tin as the two metals to be used in taking casts, but other metals are equally available provided the one poured upon the plaster cast, be a little more difficult of fusion than the one which is afterwards to be poured upon it. Zinc and lead, zinc and tin, type metal, and even the fusible compound consisting of bismuth eight parts, tin three parts, lead five parts, and mercury one part, will fully answer the purpose. Nor is it impossible to take a set of metallic castings with only one metal, provided the temperature be little higher than the point of fusion when the latter half of the casting is effected, and provided, moreover, that the part first cast, be well coated with whiting and set in a cold water bath. But whatever may be the metal used, after the metal first employed has been poured upon the plaster cast and cooled, it must be removed from the ring together with the shelf, and the plaster carefully separated from the metal. Cover this cast with whiting or smoke from a lamp as heretofore described, and when thoroughly dried restore it to its original position in the ring without the shelf. Pour upon this the melted metal to be used for the other part of the casting, and you will have a complete set, between which your plate of gold is to be swedged to its proper form.

92. When both sets of models have been thus provided, and both the gold plates prepared upon them as was fully described in a former chapter, the presence of the patient is required in order to try the plates into the mouth and ascertain whether the fit be perfect. After the plates are made to rest equally upon all parts of the subjacent gum, and while the patient remains, the dentist should adjust softened beeswax upon both the plates in the exact position which the artificial teeth are ultimately to assume. For the purpose of softening his wax on this and other occasions, he will need a small alcoholic lamp which any tin plate worker may construct in the following manner:—

This lamp which may be about two and a half inches in height and diameter, with the orifice of the tube not larger than the barrel of a common goose-quill, differs in nothing from a lamp for burning oil, excepting that it should have no orifice but that into which the wick is inserted, lest the vapour of alcohol within the vessel should be ignited. An alcoholic lamp is very useful, nay, almost indispensable to a dentist in all those months of the year when he has no fire in his laboratory; for with a cheap and simple apparatus he may dry his plaster casts, warm or melt wax, heat water for various purposes, and boil his plates in acid when he finds himself hurried in cleaning them. For drying casts, he needs only a tin cylinder a little less in diameter than his lamp, like a cup without a bottom, and cut at both ends as follows:—

Upon this cylinder when placed upon his lamp, he may set a tin skimmer as in the following cut:—

The serrated form of the ends of the cylinder answers a double purpose; it gives free circulation of the air at the bottom, and free egress to the flame of the lamp at the top. If a plaster cast be laid upon the skimmer, it becomes dry in the same manner as when placed over a fire or in an oven. When wax is to be softened for the purpose of taking models of the mouth, a vessel of tin, copper or earthen may occupy the place of the skimmer; and when plates are to be boiled in diluted acid, a vessel of copper or earthen may be employed in like manner.

93. When the beeswax has been adjusted on the plates so as to occupy in the mouth the same position which the teeth are desired to assume, taking care that the lips have just the desired support, the face its proper length, and the profile its natural outline, the operator should mark upon the wax in front the exact centre of the mouth by a perpendicular line drawn from the upper to the lower plate, whereupon the whole may be carefully withdrawn from the mouth. The patient may now be dismissed provided the size and colour of the teeth have been satisfactorily determined; and the operator may proceed to arrange the teeth, one by one, in such a manner as that their enamelled surfaces shall be level with the outer surface of the wax, and their cutting edges of the same altitude as the wax. The most convenient instrument for imbedding the teeth in the wax, is a very delicate chisel not wider than a small lateral incisor, and very thin and sharp. An alcoholic lamp may be used to keep the chisel and the wax at the proper temperature.*

94. All the teeth of both jaws being accurately arranged in the manner just described, which implies that each tooth has been ground exactly to fit the plate and the model of the gum, the whole arrangement of each jaw including the teeth, the plate, and plaster model reserved for this purpose must be secured firmly by the mixture of plaster, sand and charcoal already described, so that when the wax shall be removed from the backs of the teeth by the aid of warm water or simply with the chisel or penknife, the other parts will be firmly consolidated into one mass. Inasmuch as the process of grinding the teeth in adjusting them to the plate, is an important part of the operation of setting them on gold, I shall introduce to the notice of the student and of the profession at large, a new and elegant grinding apparatus which I have employed a skilful artist to construct, it being an adaptation of a machine originally constructed by the same artist for simply sharpening the cutting implements of a wood engraver of this city. The transformations which I have suggested, consist in adding to the wheels for sharpening small edge-tools such as a dentist constantly uses in his practice, other wheels for grinding teeth, and also for circular brushes and burnishing wheels

* The studs may be adjusted to the backs of the teeth either at this time, or, as I esteem the better method, afterwards as will be described. It is a good rule to arrange the lower set first, as good taste may direct; then let each eye-tooth of the upper jaw articulate between the ends of the canine and first bicuspid of the lower jaw. This being arranged, all the other teeth will fall into their proper places.

as well as drills.* These improvements render it one of the most perfect, available and ornamental pieces of machinery that has probably ever been offered to the dental profession in any country, and as no patent will confine its manufacture to any individual, it may be constructed in any part of the world where any good machinist is willing to incur the expense of providing himself with the models necessary for all the castings.

Y Y is a frame-work of mahogany firmly put together, each piece of which should be one and a half inch square.

* Important changes have also been made in the form of the largest wheel, and also in having two copper wheels of different thicknesses for sharpening edge tools. The hand crank also is so constructed that it may be adjusted to either end of the axle of the balance wheel, and a foot crank may be substituted if desired.

A is a cast iron wheel seventeen inches in diameter, solid throughout, being three-fourths of an inch thick near the axis and at the circumference, having a groove for the band one-fourth of an inch in breadth and depth. The general thickness of this wheel is three-eighths of an inch. The dimensions of this wheel require that the frame-work should be twenty inches in height, twenty-four inches in length, and five inches in breadth.

B is a polished brass wheel 10 inches in diameter and three-fifths of an inch thick at the circumference, formed in all other respects like the other.

C is a brass burr two inches in diameter and half an inch in thickness, having a groove like the wheels. Both the wheels revolve upon axles of steel, that of the large wheel being eight inches long and three-fourths of an inch in diameter. D and E are composition wheels for grinding teeth, composed of emory and shellac. These wheels are of various forms suited to the uses for which they are designed. They are sustained by brass chucks attached by screw-joints to the mandril of the copper wheels F G. These latter which are used for grinding dental cutting instruments are three and a half inches in diameter and of different thicknesses, the one half, the other a fourth of an inch. These copper wheels play in a semi-cylindrical box of brass, and have a rest of the same metal to support the instrument when grinding, on the side nearest the operator—and a brass band one inch in width on the opposite side to confine the paste of sweet oil and crocus used on the circumferences of the wheels instead of emory.

To keep the whole neat and clean no water should be used on the composition wheels when grinding teeth. In each end of the mandril on which the grinding wheels are suspended, is a cylindrical hole half an inch deep for the insertion of drills and brooches.

Brush wheels and burnishing wheels may be substituted for the grinding wheels, by using an instrument of the following form to prevent the waste of the emory, crocus, rotten stone, pumice stone, whiting, or other substance used for polishing plates of gold, or dental instruments.

This instrument from the point N upwards may be of tin plate one foot in diameter, and three inches in thickness, having an opening of four inches on one side, and may be lined with cotton to prevent injuring a gold plate in case it escape from the fingers of the operator. The pedestal may be of lead with a wooden shaft entering the tin cylinder at N. This machine grinds and sharpens the most delicate edge-tools in the nicest manner; carries stones for grinding mineral teeth, wheel brushes for polishing plates and drills for various purposes. The power of the multiplying wheels is such that the wheels at D E F and C revolve forty-eight times during a single revolution of the crank at H.

For all practical purposes it will be found that the hand-crank is preferable to a foot treddle for the dentist; if for no other reason excepting that he can more conveniently retard, accelerate, and arrest the motion. But there are other reasons, among which I may mention that the whole machine is more portable and compact, taking up less room in the dentist's laboratory than if it were propelled by the foot instead of the hand. I have contracted with the manufacturer of this machine to furnish them to immediate applicants at fifty dollars, exclusive of my commission for boxing, directing, and transmitting them to order.

95. After an apology for this digression, I return to the immediate subject of discourse, and proceed to state that the two parts of our double set are now ready for the adaptation of gold studs or backs to the teeth. To this end each tooth must be taken carefully from its position and provided with a gold back by means of which it is finally to be attached to the principal plate, as I have recently adopted a method of constructing these *studs* or *linings* to the teeth, which surpasses both in strength and beauty any which I have ever before seen, I take great pleasure in offering it to my brethren as one of the few improvements which I have been able to add to the common stock of dental knowledge since my connection with the profession. Instead of forming the backs of the teeth of flat plates of gold, let the plate be formed into the longitudinal section of a cylinder in the following manner; take a piece of gold plate large enough for the lining of a tooth, and placing it upon a solid block of lead, swedge it into the following form by means of a rod of steel struck by a hammer.

Then with a file remove part of the gold till it assumes the form of the cut annexed:

This when seen in the direction of the radius of the curve will appear as follows:

And when inserted upon the tooth will have the following aspect:

In all the foregoing cuts the convexity of the arch has been greater than it should be in practice, where the curve should not exceed the sixth part of a circle. This parabolic form of the outline of the stud, is the most beautiful to be imagined, and can be attained in practice only by such a section of a cone or cylinder as I have just described. When the teeth are long the superior beauty of this description of linings is particularly manifest, and even when the teeth are short, it is equally practicable. I have found that most of the platina pivots in Alcock's and Stockton's teeth, have sufficient length to fit them for this mode of mounting; but I deem it just to remark that very few of their pivots are either too long or too large; the fault being in general on the other side.

The superior strength of this method of attaching the studs to the principal plate, will be discovered at a single glance, and a much smaller quantity of solder than is commonly employed will effect the object. The small concavity between the convex stud and the tooth may be filled with gold foil in the manner of plugging teeth for caries, before re-adjusting the tooth in its place for soldering. When all the teeth have been thus provided with studs or linings, and replaced in their respective beds in the plaster, a small quantity of plaster should be applied to their points in order to secure them in their position. A piece of solder should be placed on the lining of each tooth to cover both the platina pivots and resting on the principal plate.

96. Inasmuch as it may not be convenient for every student to procure that species of self-acting blowpipe which was described in a former chapter, I shall here introduce the drawing of one which I have recently constructed for my own use, and which operates as perfectly as I could desire.

A is a vessel of thick brass four inches in diameter and three in height, having a tube at E extending above the lid half an inch and within the vessel two inches, and having also at H a pipe extending within the vessel almost to the top and attached firmly to the pipe E in order to keep it in its place. The external orifice of the pipe H should be a little larger than that of the common blowpipe and it would be best if tipped with platina. This upper vessel is attached to the standard D which is an iron rod one-third of an inch in diameter, and eighteen inches in height, by means of a brass cylinder L having partitions near each extremity, through an orifice in each of which the rod passes. Within this cylinder is a spring of hammered brass plate slightly curved, which presses against the rod and preserves the vessel A at any desired position. The manner in which the spring presses upon the rod may be seen in the following cut, and when the vessel is removed from the rod, the spring may be readily withdrawn from the cylinder. The rod D is firmly attached to a platform C six inches in diameter and three-fourths of an inch thick of solid lead.

The vessel B is an alcoholic lamp of the same dimensions as that above it, having two pipes upon the top each reaching to the centre of the lamp within, and projecting one inch above it. It has also a spout at K, the external orifice of which may be nearly as high as the lamp itself and half an inch in diameter. The pipes E of the upper vessel and G of the lower are used for introducing alcohol, while the pipes F and K are to be provided with common lamp-wick. When both the vessels are supplied with as much alcohol as each will readily receive, the whole apparatus is fit for use; but care must be taken that the tubes E and G be kept well corked; and all the tubes should have brass caps to prevent the evaporation of the spirits when the lamp is not in use, and also to give a finish to the apparatus. When the blaze of the wick at F heats the alcohol in the upper vessel, the liquid is converted into vapour which is instantly propelled through the orifice of the pipe H, and if this orifice be brought to the proper position, the stream of alcoholic vapour will of itself when ignited produce a beautiful jet of flame; but if the wick at K be also ignited, the flames of both combined will be found sufficient for all the ordinary purposes of a dentist, and will melt his lead and tin for castings in a few seconds of time. Although Mr. J. Parmly, who was the first dentist among my acquaintances who applied the self-acting blowpipe to dental purposes by supplying the lamp with two separate wicks instead of one as in Hook's blowpipe, has been in the habit of using it only for pieces of plate-work when enveloped in plaster and then only after heating the whole to redness in a furnace, yet I am satisfied from my own experience that a lamp of the dimensions just described, when properly managed, may be made to solder any piece of plate-work in a very few minutes without previously heating it. But to effect this to advantage, the piece to be soldered should be placed in a block of pumice stone excavated for the purpose, inasmuch as charcoal under the action of the alcoholic flame, gives off so many sparks as to render it unfit for use. One evident advantage of this kind of blowpipe is that the right hand of the operator is at liberty to direct the solder at his pleasure by means of a rod of platina wire of small size, three inches in length and soldered to a similar wire of silver or brass eight inches in length, as follows:—

When the platina wire is hotter than the gold plate, the solder will attach itself to the platina, and *vice versa;* so that the artist has it in his power by the skilful use of this little rod to transfer the melted solder to any part of the plate where it may be needed most, or indeed to take away any excess altogether; inasmuch as he can impart alternately the greater quantity of heat to the plate or to the rod at his pleasure. When pumice stone of sufficient size cannot be conveniently obtained to support the piece to be soldered, a small iron shovel like the annexed figure, will be found convenient.

The sides of this shovel are turned up to the height of about an inch in order to secure the piece in its position, and a part of the right side is cut away to afford ready access to the flame. The blade may be two inches square and the handle nine inches in length; the former of sheet iron, the latter of large iron wire.

75. I have purposely reserved a consideration of the springs and their appurtenances by which double sets are usually retained in their position, for another chapter, although the contrivances for attaching the springs to the two plates on either side of the mouth, are sometimes soldered to the plates before the teeth are ground to their places. Yet as the time when they are connected with the plates, has nothing to do with their general construction, I have thought it preferable to describe these parts in all their varieties in a separate chapter.

I ought perhaps to offer an apology for having interrupted the history of the construction of double sets by introducing protracted descriptions of the new grinding apparatus and the improved blow-pipe; but as utility is my object rather than the graces of composition; and as I am confident that the two implements thus minutely described in this chapter, will prove of essential benefit to the profession, I submit them to the test of experiment. For myself I would not willingly be without them for ten times their cost.

[TO BE CONTINUED.]

## Figures and Tables

**Figure f1:**
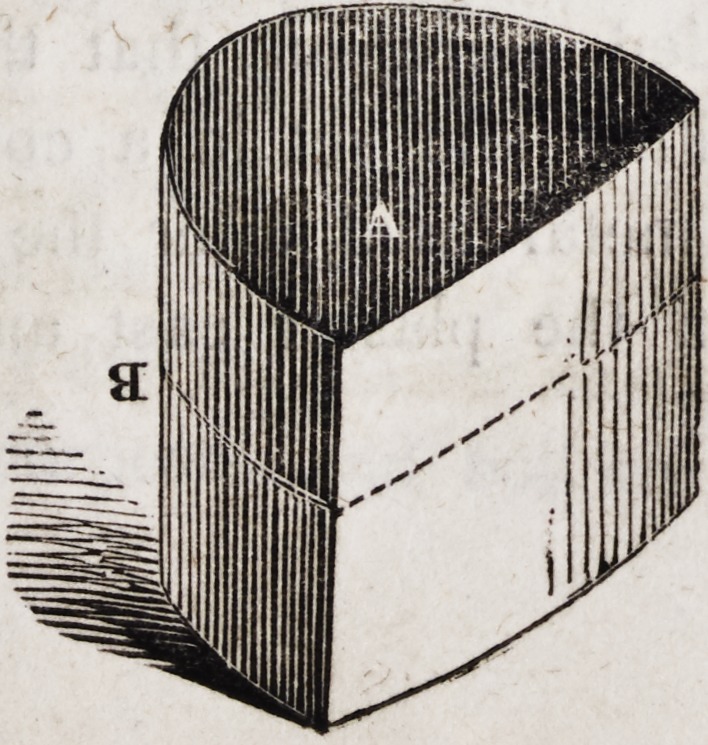


**Figure f2:**
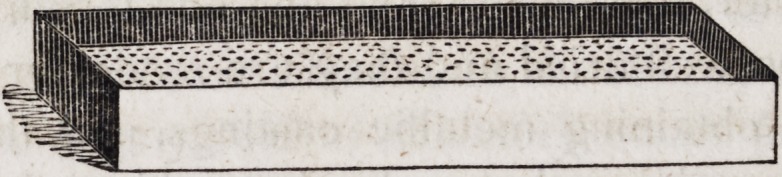


**Figure f3:**
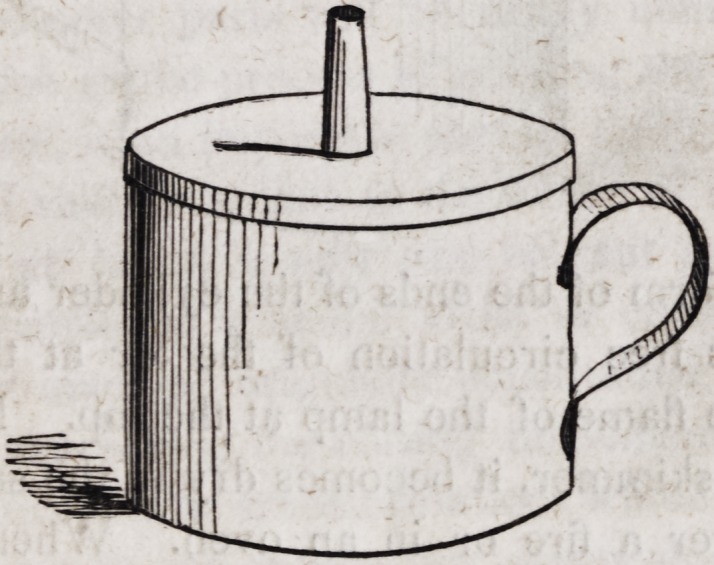


**Figure f4:**
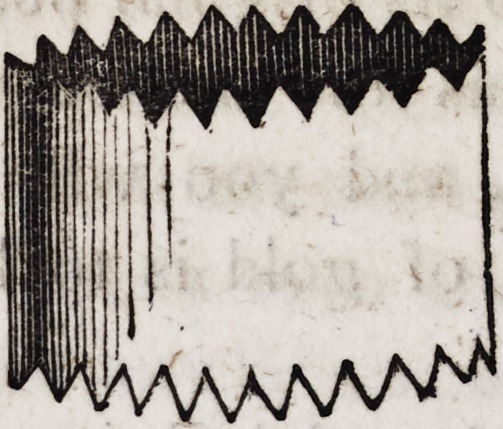


**Figure f5:**
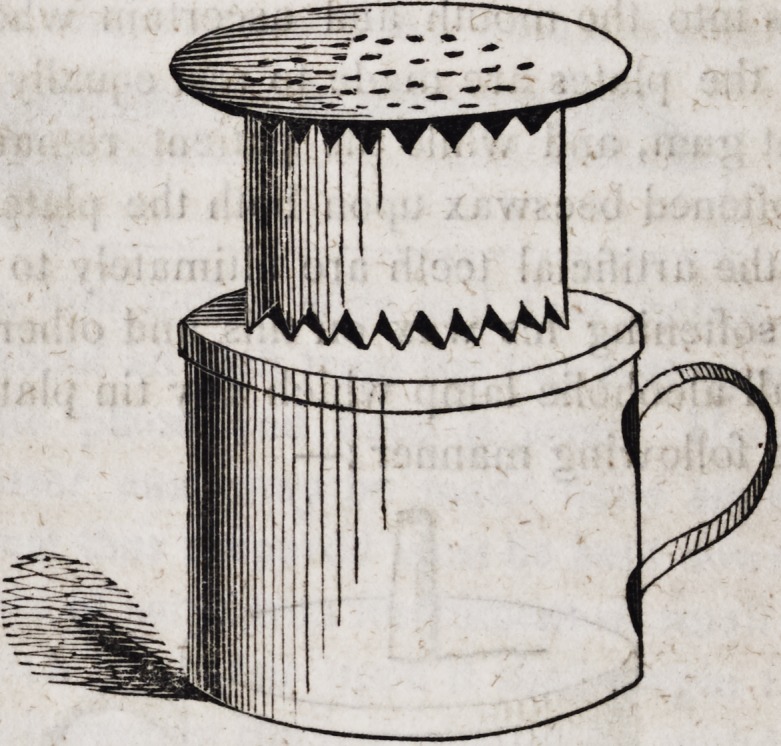


**Figure f6:**
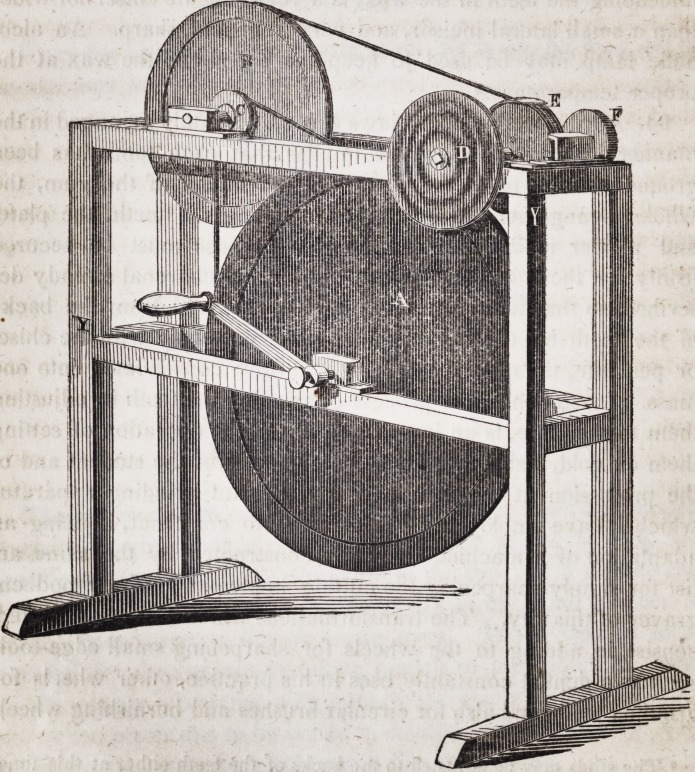


**Figure f7:**
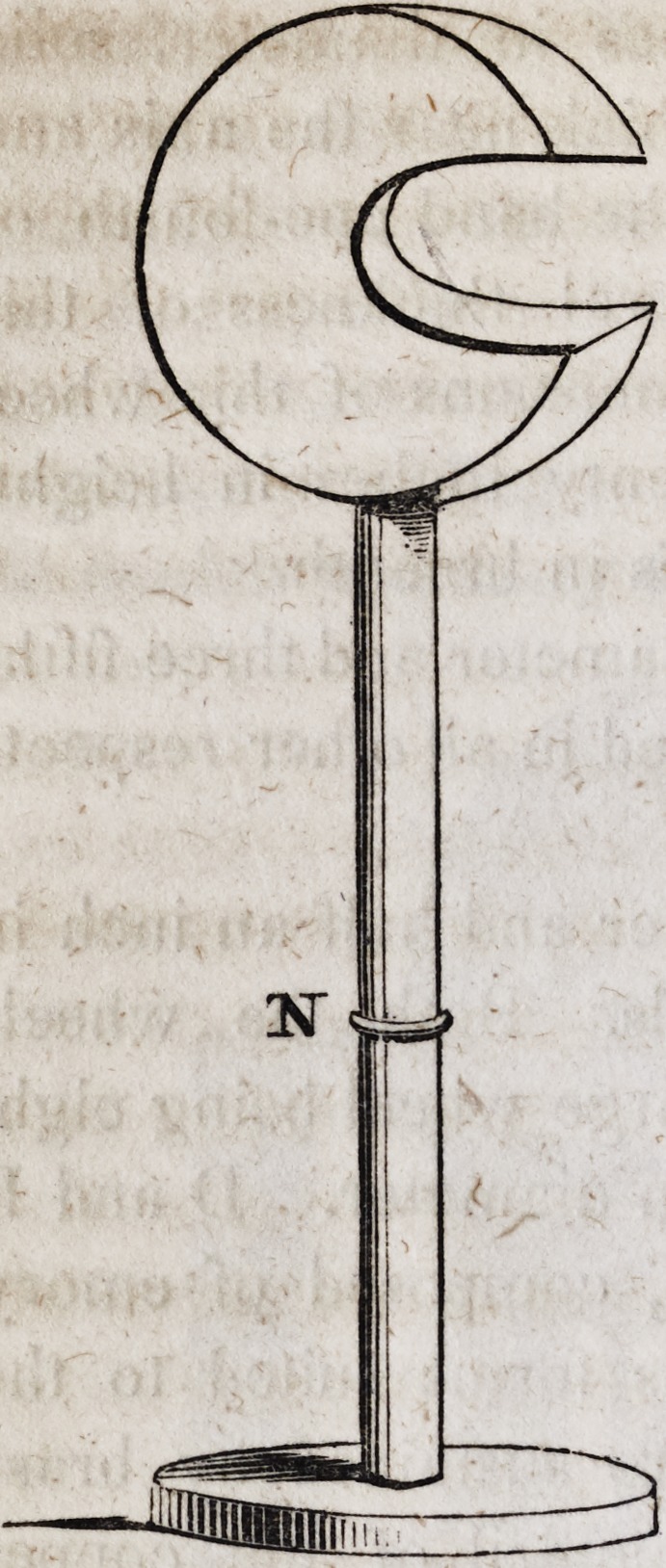


**Figure f8:**
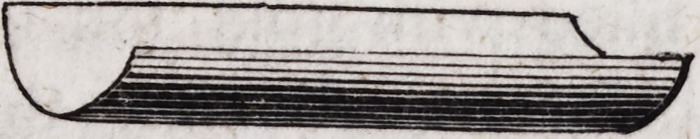


**Figure f9:**
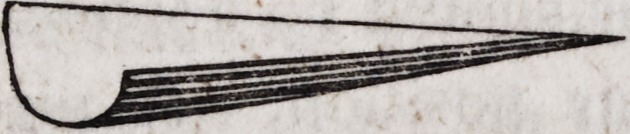


**Figure f10:**
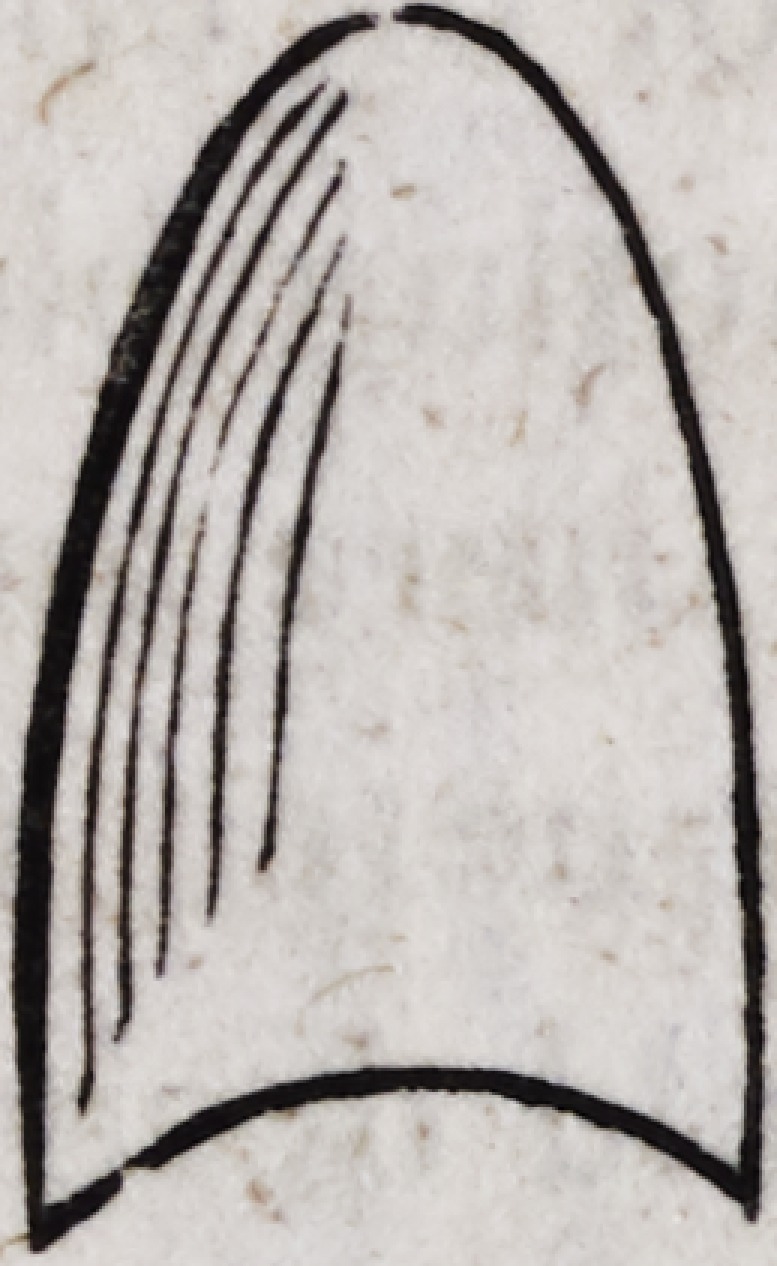


**Figure f11:**
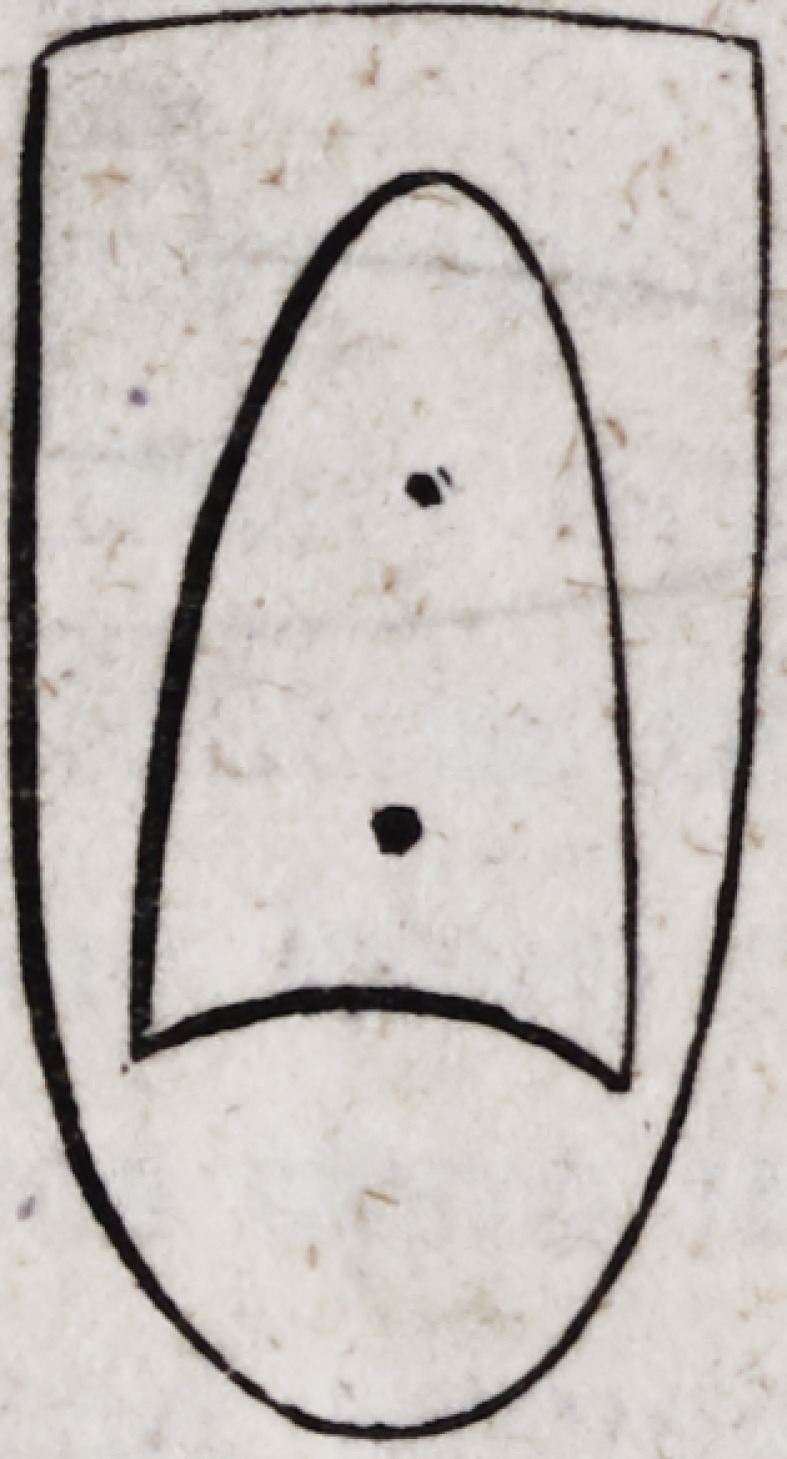


**Figure f12:**
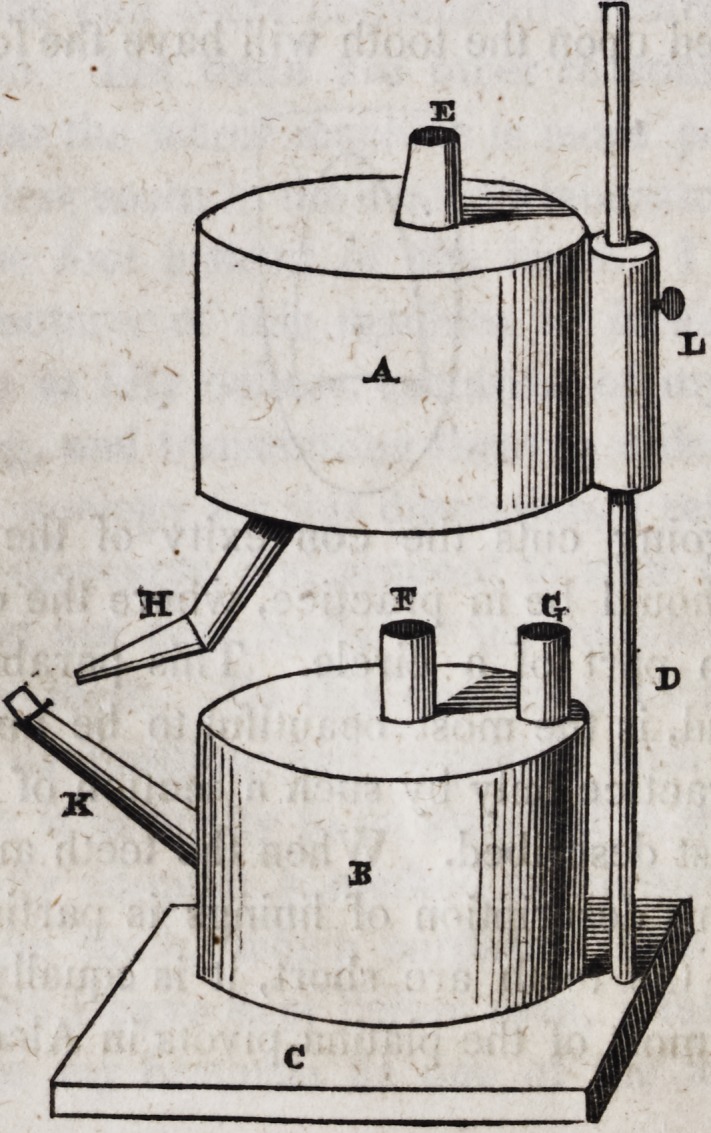


**Figure f13:**
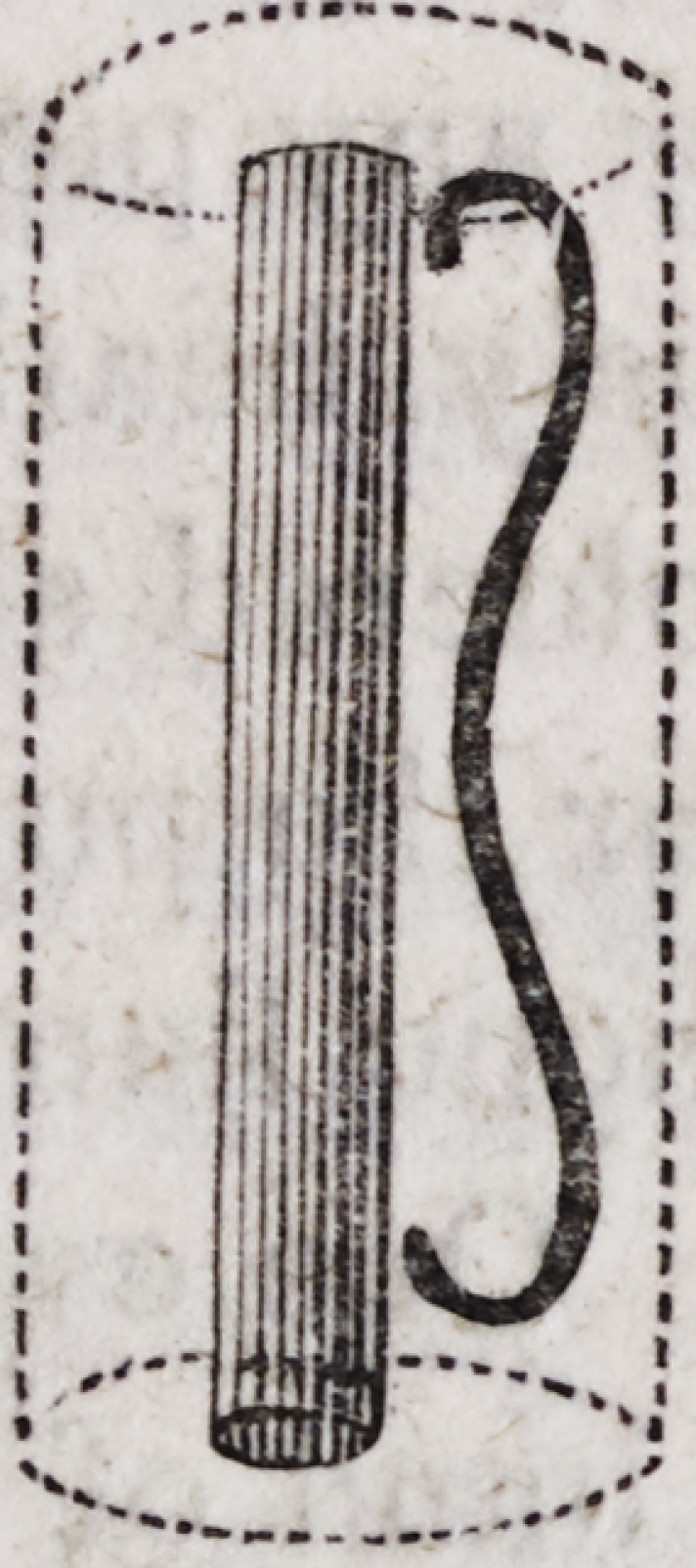


**Figure f14:**
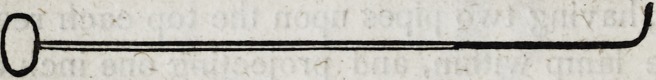


**Figure f15:**